# Radiological screening of maternal periodontitis for predicting adverse pregnancy and neonatal outcomes

**DOI:** 10.1038/s41598-020-78385-0

**Published:** 2020-12-04

**Authors:** Ju Sun Heo, Ki Hoon Ahn, Jung Soo Park

**Affiliations:** 1 Department of Pediatrics, Anam Hospital, Korea University College of Medicine, Seoul, Republic of Korea; 2 Department of Obstetrics and Gynecology, Anam Hospital, Korea University College of Medicine, Seoul, Republic of Korea; 3 Department of Periodontology, Anam Hospital, Korea University College of Medicine, Seoul, Republic of Korea

**Keywords:** Diseases, Medical research

## Abstract

It is well known that periodontitis, diagnosed mainly by periodontal probing, is associated with adverse pregnancy outcomes. However, periodontal probing is time-consuming, highly discomforting, inaccurate, and invasive. We aimed to assess whether periodontitis severity based on radiological staging in accordance with the 2017 new consensus classification was related to adverse pregnancy and neonatal outcomes. The medical records of 165 mothers who underwent panoramic radiography within 5 years before and after the time of delivery and of their singleton neonates were retrospectively reviewed. Twenty-two mothers (13.3%) had severe periodontitis (SP), and 143 (86.7%) had mild or moderate periodontitis (MP). In relation to adverse pregnancy outcomes, uterine leiomyoma (18.2% vs. 4.2%, *P* = 0.029), chronic hypertension (9.1% vs. 0.7%, *P* = 0.047), and preeclampsia (13.6% vs. 2.1%, *P* = 0.032) occurred significantly more frequently in the SP group than in the MP group. The incidences of very preterm birth (13.6% vs. 1.4%, *P* = 0.017), extremely preterm birth (9.1% vs. 0.7%, *P* = 0.047), and small for gestational age (22.7% vs. 5.6%, *P* = 0.017) were also significantly higher in the SP group than in the MP group. Radiological screening of maternal periodontitis could be useful for predicting adverse pregnancy and neonatal outcomes as well as diagnosing SP in pregnant women.

## Introduction

Periodontitis is a chronic inflammatory disease that occurs in response to a disease-associated multispecies bacterial community and results in the destruction of tooth-supporting connective tissue and bone^1,2^. It is a worldwide health problem, and the sixth most common disease globally^3^. In Korea, the National Health Insurance Service recently surveyed the number of patients with periodontal disease from 2012 to 2016^4^. The prevalence was reported to have increased from 8.34 million patients in 2012–14.19 million patients in 2016, with a 17% increase per year.

Periodontitis has been linked to adverse pregnancy outcomes such as preterm birth, low birth weight, and preeclampsia^5–12^. Considering these results, it seems essential for physicians or gynaecologists to recommend dental screening for their patients when planning to conceive a baby or even during pregnancy. However, in the clinical field, consultants for regular dental check-ups for women of childbearing age or pregnant mothers are not properly being performed largely due to maternal resistance and lack of public knowledge on the necessity. Periodontal screening could be burdensome, especially in pregnant women, due to the invasiveness and complexity of the whole procedure. Clinical probing measurement is widely accepted as a basic method to diagnose the presence and severity of periodontitis. Periodontal probing is performed by inserting a blunt instrument into a gingival crevice (narrow space between the tooth surface and free gingiva) until it meets certain resistance. The examiner needs to locate the base of the junctional epithelium (bottom of the sulcus) precisely to obtain medically reliable and consistent measurements^13^. Major variables generally used to diagnose the “disease” are periodontal pocket depth and clinical attachment level (CAL), and full-mouth examination is often unavoidable for reliable diagnosis. Therefore, the whole procedure is quite time-consuming, cumbersome, and discomforting for patients. In addition, probing measurement is, although valuable, it may be, always subject to inter-examiner and intra-examiner measuring errors, which can range up to 1.07 mm according to previous reports^14^. Even after proper measurement, it is still confusing to determine whether to treat the disease because of the variety of diagnostic criteria used in various research articles^15,16^. Finally, probing through untreated periodontal tissues harbouring significant amounts of pathogenic microbes can cause systemic bacteraemia, which elevates the risks of adverse pregnancy outcomes^17^.

These considerations imply the necessity of a standardised, straightforward, and non-invasive periodontal screening tool that can direct high-risk patients who would require close periodontal monitoring to reduce the risk of adverse pregnancy outcomes. In the novel staging system developed in 2017, radiographically recognisable parameters, such as the amount of bone loss, tooth loss, and furcation involvement, were newly introduced in severity determination, thereby suggesting possible standardisation in radiologic classification^18^. However, there has been no previous study on the association between the severity of periodontitis diagnosed using this tool and adverse pregnancy/neonatal outcomes.

This study aimed to assess whether the severity of maternal periodontitis classified by radiological screening in accordance with the 2017 consensus classification was related to adverse pregnancy and neonatal outcomes.

## Results

### Maternal characteristics and morbidities in the severe periodontitis (SP) and mild/moderate periodontitis (MP) groups

Of the 165 mothers enrolled, 22 (13.3%) had SP and 143 (86.7%) had MP. Table 1 shows the baseline characteristics and morbidities of the mothers according to the severity of periodontitis. Maternal age, pre-pregnancy body mass index (BMI), and incidence of caesarean section were higher in the SP group than in the MP group. However, the difference was only marginally significant (*P* < 0.1). Uterine leiomyoma, chronic hypertension, and preeclampsia occurred significantly more frequently in the SP group than in the MP group. After adjusting for maternal variables including maternal age ≥ 35 years, pre-pregnancy BMI, and preeclampsia, maternal SP was associated with an increased risk of uterine leiomyoma (adjusted odds ratio [aOR] 5.555, 95% confidence interval [CI] 1.371–22.503, *P* = 0.016). Other factors, including weight gain during pregnancy, prior preterm birth, preterm labour, chorioamnionitis, and chronic medical diseases, were not significantly different between the SP and MP groups.

**Table 1:** Maternal baseline characteristics and morbidities.

Factors	Severe periodontitis n = 22	Mild/moderate periodontitis n = 143	*P*-value
Age (year)	34 (30, 38)	31 (29, 34)	0.068
Age ≥ 35 years	8 (36.4)	34 (23.8)	0.291
Nulliparous	9 (40.9)	81 (56.6)	0.177
Height (cm)	163 (158, 167)	161 (157, 165)	0.267
Pre-pregnancy weight (kg)	56 (52, 66)	54 (50, 58)	0.121
Pre-pregnancy body mass index (kg/m^2^)	21.5 (20.1, 24.2)	20.8 (19.3, 22.3)	0.092
Weight gain during pregnancy (kg)	12.5 (8.7, 15.3)	13.0 (10.5, 16.1)	0.378
Body mass index change during pregnancy (kg/m^2^)	5.1 (3.2, 5.6)	5.0 (3.9, 6.1)	0.301
In-vitro fertilization	1 (4.5)	4 (2.8)	0.516
Cesarean section	16 (72.7)	73 (51.0)	0.068
Prior preterm birth	2 (9.1)	5 (3.5)	0.235
Preterm labor	3 (13.6)	20 (14.0)	1.000
Prelabor rupture of membrane > 18 h	0 (0.0)	16 (11.2)	0.134
Chorioamnionitis	0/11 (0.0)	3/56 (5.4)	1.000
Funisitis	0/11 (0.0)	0/56 (0.0)	
Pelvic inflammatory disease history	0 (0.0)	1 (0.7)	1.000
Uterine leiomyoma	4 (18.2)	6 (4.2)	0.029*
Type 1 diabetes mellitus	1 (5.0)	0 (0.0)	0.125
Gestational diabetes mellitus	3 (15.0)	7 (5.0)	0.113
Chronic hypertension	2 (9.1)	1 (0.7)	0.047*
Preeclampsia	3 (13.6)	3 (2.1)	0.032*
Thyroid disease	3 (13.6)	27 (18.9)	0.768
Chronic medical disease	6 (27.3)	35 (24.5)	0.793

Values are expressed N (%) or median (interquartile range). *P < 0.05

### Neonatal characteristics in the SP and MP groups

The baseline neonatal characteristics are presented in Table 2. There were no significant differences between the groups in terms of gestational age (GA), incidence of preterm birth (GA < 37 weeks), sex, or z-scores of birth weight, height, and head circumference. However, the incidences of very preterm birth (GA < 32 weeks) and extremely preterm birth (GA < 28 weeks) were significantly higher in the SP group than in the MP group.

**Table 2:** Neonatal baseline characteristics.

Factors	Severe periodontitis n = 22	Mild/moderate periodontitis n = 143	*P*-value
Gestational age (weeks)	37^+5^ (36^+4^, 39^+5^)	38^+5^ (37^+2^, 39^+3^)	0.227
Gestational age < 37 weeks	6 (27.3)	20 (14.0)	0.122
Gestational age < 32 weeks	3 (13.6)	2 (1.4)	0.017*
Gestational age < 28 weeks	2 (9.1)	1 (0.7)	0.047*
Birth weight (g)	3175 (2238, 3528)	3220 (2880, 3440)	0.487
Birth weight z-score	− 0.196 (− 1.275, 0.454)	− 0.023 (− 0.424, 0.351)	0.345
Height at birth (cm)	50.0 (46.8, 53.0)	50.0 (48.0, 51.0)	0.497
Height at birth, z-score	0.380 (− 0.819, 1.349)	0.015 (− 0.457, 0.654)	0.249
Head circumference at birth (cm)	33.3 (31.8, 34.5)	34.0 (32.5, 35.0)	0.221
Head circumference at birth, z-score	− 0.507 (− 1.451, 0.306)	− 0.264 (− 0.915, 0.402)	0.215
Small for gestational age	5 (22.7)	8 (5.6)	0.017*
Male	15 (68.2)	78 (54.5)	0.257
Neonatal resuscitation program at delivery room	7 (31.8)	21 (14.7)	0.064
Apgar score, 1 min	9.0 (6.5, 9.0)	9.0 (8.0, 9.0)	0.279
Apgar score, 5 min	10.0 (8.8, 10.0)	10.0 (9.0, 10.0)	0.451
Neonatal intensive care unit admission	8 (38.4)	32 (22.4)	0.182
Initial white blood cell count	10,590 (7800, 16,300)	14,000 (9450, 19,100)	0.065
Initial C-reactive protein	0.16 (0.13, 0.99)	0.14 (0.11, 0.48)	0.458

Values are expressed N (%) or median (interquartile range). *P < 0.05

**Figure 1:**
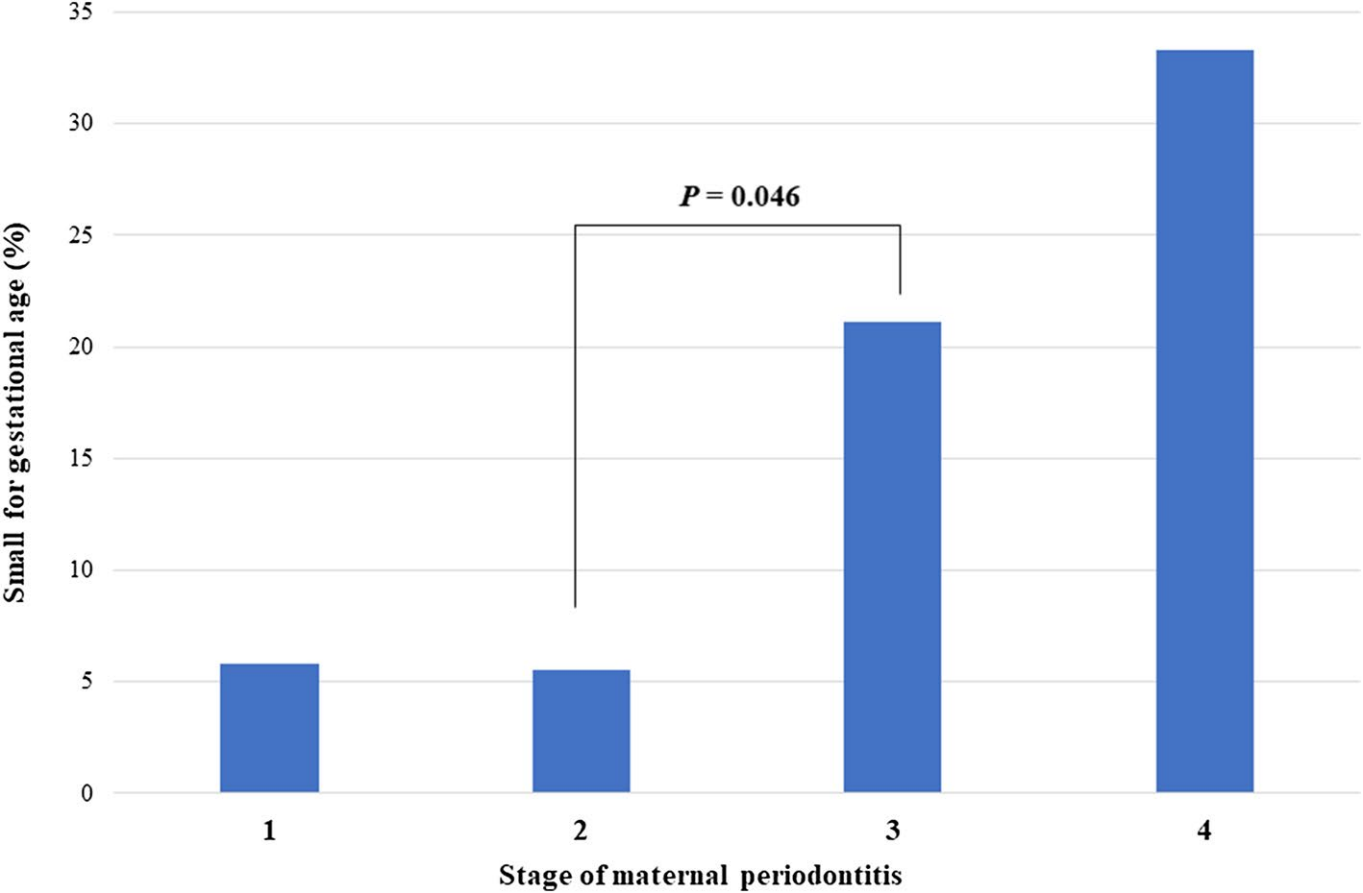
Incidence of small for gestational age (SGA) according to the stage of maternal periodontitis. The incidence of SGA remained low in those with stage ≤ II disease but increased rapidly in those with stage ≥ III disease.

The incidence of small for gestational age (SGA) was significantly higher in the SP group than in the MP group. The incidence of SGA remained low, at around 5%, in those with stage ≤ II disease but increased rapidly in those with stage ≥ III disease (Fig. 1). After adjusting for maternal and neonatal variables, including maternal age ≥ 35 years, pre-pregnancy BMI, uterine leiomyoma, preeclampsia, caesarean section, and GA < 32 weeks, maternal SP was associated with an increased risk of SGA (aOR 4.488, 95% CI 1.116–18.058, *P* = 0.035).

The initial white blood cell count was marginally higher in the MP group, but the initial C-reactive protein levels were similar between the SP and MP groups.

### Neonatal characteristics and morbidities of preterm infants

Among the 26 infants born prematurely, six (23.1%) were born to mothers with SP, and 20 (76.9%) were born to mothers with MP. Table 3 shows the neonatal baseline characteristics and morbidities of preterm infants according to their mothers’ severity of periodontitis. Infants in the SP group were born at an earlier GA than infants in the MP group, but this difference was not significant. The incidence of very preterm birth was borderline significantly higher in the SP group than in the MP group. Infants in the SP group not only had lower birth weights but also lower z-scores of birth weight. There was no significant difference in height or head circumference. The incidence of SGA was higher in the SP group than in the MP group, but this difference was not significant. The duration of respiratory support was significantly longer and the incidences of retinopathy of prematurity (ROP) and treated patent ductus arteriosus (PDA) were significantly higher in the SP group than in the MP group.

**Table 3:** Neonatal baseline characteristics and morbidities among the preterm infants.

Factors	Severe periodontitis n = 6	Mild/moderate periodontitis n = 20	*P*-value
Gestational age (weeks)	32^+2^ (28^+3^, 36^+2^)	34^+5^ (33^+5^, 36^+3^)	0.268
Gestational age < 32 weeks	3 (50.0)	2 (10.0)	0.062
Gestational age < 28 weeks	2 (33.3)	1 (5.0)	0.123
Birth weight (g)	1475 (845, 2393)	2300 (2040, 2896)	0.046*
Birth weight z-score	− 1.166 (− 1.461, 0.400)	0.251 (− 0.231, 0.539)	0.083
Height at birth (cm)	39.8 (33.8, 49.5)	46.3 (44.2, 48.9)	0.157
Height at birth, z-score	− 0.249 (− 1.846, 0.926)	0.549 (− 0.119, 1.235)	0.242
Head circumference at birth (cm)	27.8 (23.6, 33.3)	31.3 (30.1, 33.9)	0.219
Head circumference at birth, z-score	− 0.642 (− 1.714, 0.597)	0.431 (− 0.639, 0.887)	0.176
Small for gestational age	2 (33.3)	1 (5.0)	0.123
Male	3 (50.0)	10 (50.0)	1.000
Neonatal resuscitation program at delivery room	5 (83.3)	7 (35.0)	0.065
Apgar score, 1 min	3.0 (1.8, 7.3)	8.0 (7.0, 9.0)	0.003*
Apgar score, 5 min	8.0 (6.0, 9.3)	9.0 (9.0, 10.0)	0.062
Initial white blood cell count	6690 (4325, 11177)	9050 (7843, 12775)	0.108
Initial C-reactive protein	0.16 (0.09, 0.99)	0.13 (0.40, 0.24)	0.933
Sepsis	0 (0.0)	0 (0.0)	
Respiratory distress syndrome	3 (50.0)	2 (10.0)	0.062
Moderate to severe bronchopulmonary dysplasia	1 (16.7)	0 (0.0)	0.231
Duration of respiratory support (days)	18.5 (0.0, 77.3)	0.0 (0.0, 1.5)	0.046*
Duration of total parenteral nutrition (days)	5.5 (0.0, 59.3)	0.0 (0.0, 0.0)	0.176
Retinopathy of prematurity	3 (50.0)	0 (0.0)	0.008*
Treated retinopathy of prematurity	2 (33.3)	0 (0.0)	0.046*
Treated patent ductus arteriosus	2 (33.3)	0 (0.0)	0.046*
Necrotizing enterocolitis ≥ stage 2	2 (33.3)	1 (5.0)	0.123
Intraventricular hemorrhage	1 (16.7)	3 (15.0)	1.000
Isolated intraparenchymal hemorrhage	1 (16.7)	0 (0.0)	0.240
Periventricular leukomalacia	0 (0.0)	0 (0.0)	

Values are expressed N (%) or median (interquartile range).*P < 0.05

## Discussion

This study presents a novel finding that radiological screening of maternal periodontitis can predict adverse pregnancy and neonatal outcomes.

According to a recent review article that systematically analysed the prevalence of periodontitis using data from a total of 291,170 individuals from 37 nations, SP affected 10.8% of people aged 15–99 years worldwide. Even after age standardisation, the prevalence of SP was 11.2% in the population, and this value has remained unchanged over the previous two decades^3^. The prevalence of SP in our study was 13.3% among 165 pregnant women aged between 19 and 42 years. Of these, 42 (25.5%) were older than 35 years. The characteristics of our subject pool seem to fit the characteristics of the generalised population, based on the similarity in the disease prevalence rate.

In our study, we applied the newly established 2017 consensus classification that introduced a staging system, as in oncology, that incorporated additional ideas and concepts other than the amount of tissue destruction. This new system includes additional aspects per category besides disease severity, such as treatment complexities and prognosis assessment^19^. Therefore, stage I or II periodontitis not only differ from staged III or IV periodontitis in terms of disease severity but also show different disease trajectories. Thus, those who present with stage III or IV periodontitis are likely to respond unpredictably to standard periodontal therapy, which focuses on reducing the bacterial burden in gingival crevices^20^.

The general idea is that a CAL ≥ 3 mm in the interproximal region of at least two different and non-adjacent teeth constitutes a diagnosis of periodontitis. If only radiological bone loss is used for assessment without considering CAL, underdetection of incipient periodontitis would be likely to occur. A substantial portion of surrounding buccal or lingual side tissues needs to be affected by inflammatory bone loss before it can be visualised by conventional radiography. Therefore, determining whether a patient has no periodontitis or stage I disease is mostly impossible if only radiography data are available for making a diagnosis. However, differentiation between stage I or II cases and stage III or IV cases is possible even with radiography data alone^19^ (see Supplementary Fig. S1 online). Our focus is on identifying SP, which might be associated with genetically impaired host immune response or chronic medical status, resulting in adverse pregnancy and neonatal outcomes^21^.

Although some studies have reported a positive association between periodontitis and SGA^16,22^, this association has been inconsistent among studies^15,23^. In this study, the incidence of SGA in the SP group was 22.7%, which is about two times higher than the prevalence in the general population of South Korea^24^. Maternal SP was associated with an increased risk of SGA, even after adjustment for the risk factors of SGA. This pattern was also observed in preterm infants. The z-score of birth weight was lower in the SP group than in the MP group, with borderline significance. There are two possible mechanisms to explain this association. First, periodontitis is a chronic inflammatory disease that acts as a reservoir for anaerobic gram-negative microorganisms, lipopolysaccharides (LPS), and cytokines, including interleukin-1β, prostaglandin E2, and tumour necrosis factor (TNF)-α^25^. This systemic inflammatory response may target the placental membranes via the bloodstream^26^, which ultimately results in abnormal placental or foetal development that impacts foetal growth^16^. A second possible mechanism involves foetal growth restriction due to excessive glucocorticoids. Placental 11β-hydroxysteroid dehydrogenase 2 (HSD2), a glucocorticoid-catalysing enzyme, protects against foetal growth restriction by preventing active glucocorticoids present in the maternal circulation from entering the foetal circulation. In a recent study, bacterial LPS was shown to downregulate 11β-HSD2 by suppressing proliferator-activated receptor-γ in placental trophoblasts^27^. This may contribute to excessive glucocorticoid production, resulting in LPS-induced foetal growth restriction. Those with SP could have increased serum levels of LPS^28^. Therefore, LPS-induced foetal growth restriction may occur by placental 11β-HSD2 downregulation in those with periodontitis.

It is well known that maternal periodontitis is related to preterm birth. However, there has been no study on the association between maternal periodontitis and postnatal complications in preterm infants. This is the first study to report this association. The incidences of ROP and treated PDA were significantly higher in the SP group. The earlier GA in the SP group might be the major cause of these results. However, it should also be considered that maternal periodontitis itself may have an effect on the incidences of ROP and PDA. Inflammation has been shown to be a key modulator of ROP development and progression^29^. This is supported by the findings of some epidemiological studies^30^. Inflammation plays an important role in the pathogenesis of PDA as well as ROP. It has been reported that chorioamnionitis is associated with the persistence of PDA^31,32^. Bacterial endotoxins and TNF stimulate the production of prostaglandin E2 in human decidua, which is the most potent mediator for the maintenance of ductus arteriosus patency^5,33^. Further clinical research is needed to prove the association between maternal periodontitis and postnatal morbidities in preterm infants.

Uterine leiomyoma, also known as uterine fibroids, is one of the most common benign tumours in women, with 70% of affected women being of reproductive age^34,35^. In this study, we concluded that uterine leiomyoma might be related to maternal periodontitis. The following two hypotheses explain this association. First, aromatase activity, a key enzyme involved in the synthesis of oestrogen from androgens, is elevated in response to chronic low-grade inflammation^36^. By stimulation of the activity of aromatase, the level of oestrogen synthesised in leiomyoma smooth cells increases, promoting cellular proliferation; this results in the development of uterine leiomyoma^37,38^ Since periodontitis is known to induce chronic low-grade systemic inflammation^5^, it is reasonable to assume that non-treated SP may have contributed to aromatase overexpression, resulting in the development of uterine leiomyomas. Second, bacterial plaques, LPS, and DNA from bacteria induce activation of activating protein-1 (AP-1) and nuclear factor-kB (NF-kB) in periodontitis patients^39^. AP-1 activity and NF-kB are also involved in cell growth in uterine leiomyomas^40,41^. This is the first study to report a link between periodontitis and uterine leiomyoma. Larger cohort studies are needed to establish more distinct clinical evidence about this correlation.

There were several limitations to this study. First, the small sample size and relative rarity of SP, especially in mothers of preterm infants, limited our statistical analyses, which were insufficient to confirm the effects of SP on the development of ROP and PDA. Despite this limitation, maternal SP was identified as a risk factor for uterine leiomyoma and SGA. Second, the data on chorioamnionitis and funisitis were imprecise, as only half of the mothers had available results of their placental biopsy. Finally, due to the retrospective nature of the study, there was a lack of clinical periodontal data that provide information on the inflammatory status of the periodontium during the examination period. However, our primary concern was to investigate a periodontally high-risk group of pregnant women who might possess an impaired host immune response or chronic medical status that would aggravate pregnancy-related complications. Radiographically identifiable extensive bone loss or tooth loss, not just acute inflammation, would be helpful in discriminating this high-risk group.

In conclusion, this study suggests that radiological screening of maternal periodontitis can predict adverse pregnancy and neonatal outcomes. Due to the radiologically distinct disparities between stage II and III periodontitis, this strategy can be widely used in the clinical field. It is simple, clinically intuitive, and can minimise the burden on pregnant patients. Our results are clinically meaningful for counseling women of childbearing age, especially women who are planning and already pregnant. The screening of the severity of periodontal diseases by radiographs and the explanation of the potential association of periodontitis with perinatal outcomes would be implicated in the regular health check-up period of women of childbearing age.

## Methods

### Study design and population

This study was conducted using a retrospective cohort design. The study subjects were mothers who underwent panoramic radiography within 5 years before and after the time of delivery in the Korea University Anam Hospital between January 1, 2010, and December 12, 2019. Panoramic radiographs were taken when the subjects had visited the Department of Dentistry, Anam Hospital for caries treatment or 3rd molar extraction. Cases involving stillbirth or multiplets were excluded. The medical records of mothers included in the study and their newborns were retrospectively reviewed.

**Figure 2:**
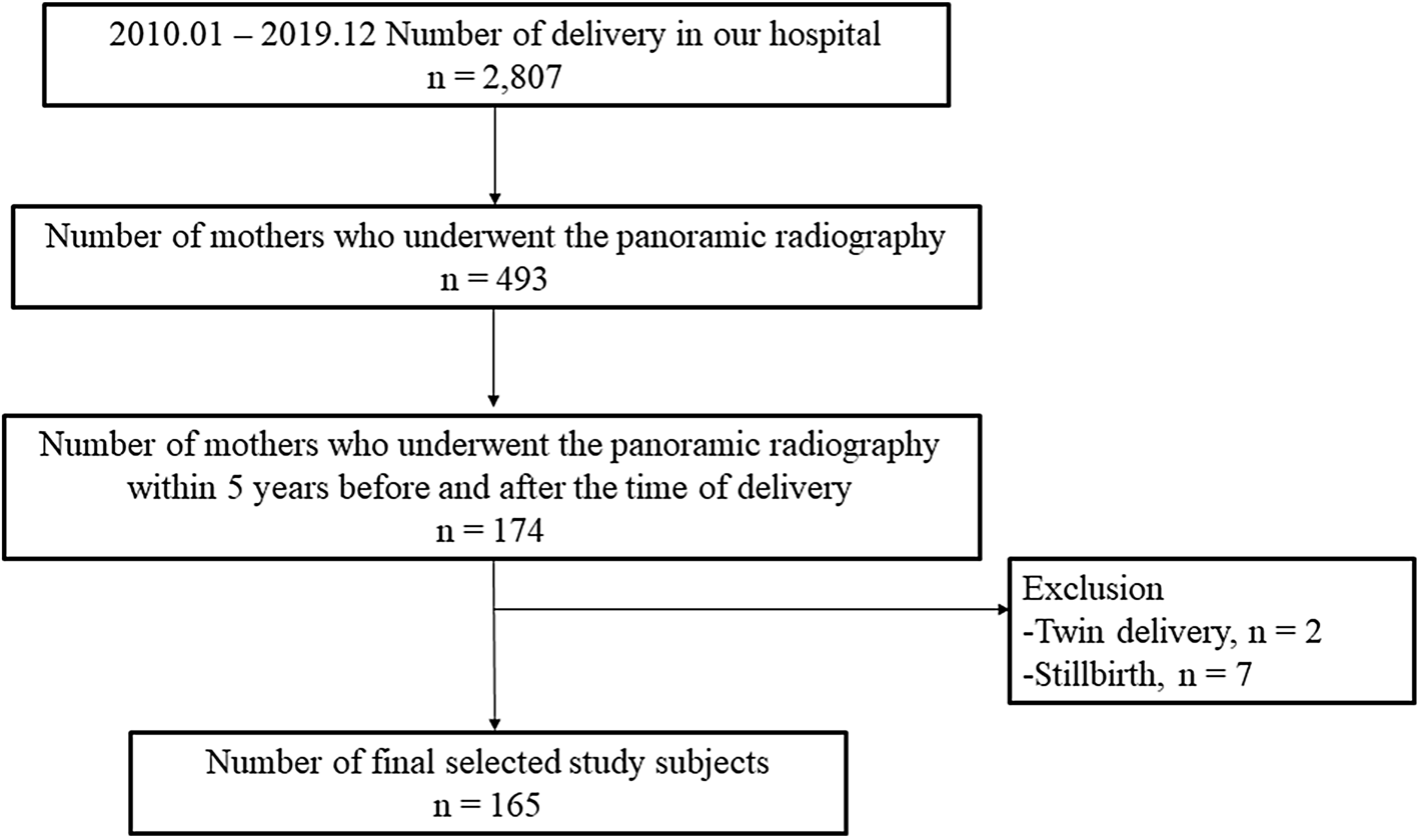
Flow chart for inclusion of the study population.

A total of 2807 mothers gave birth during the study period (Fig. 2). Among them, 493 mothers (17.6%) underwent panoramic radiography, and 174 (6.2%) underwent panoramic radiography within 5 years before and after the time of delivery. Among the 174 mothers, we excluded two mothers who gave birth to twins and seven mothers with stillbirth. A final study population of 165 mothers and their neonates was included in this study.

### Clinical data collection

We used a new classification of periodontal disease introduced following consensus reports of an International Workshop that took place in November 2017 to assign MP or SP in this study^19,42^. MP refers to stage I or II periodontitis, while SP refers to stage III or IV periodontitis. Periodontal staging was performed by a single periodontal specialist. Intra-examiner reliability was validated using kappa coefficients from 100 panoramic radiographs before the study (Cohen’s kappa value: 0.90). Cases showing radiographic bone loss that extends to the mid-third of the root or beyond, tooth loss due to periodontal causes, vertical ridge defect, or class II or III furcation involvement were assigned to the SP group (stage III or IV). Periodontally healthy cases fell into stage I because it is impossible to discern healthy cases from stage I without the help of full-mouth periodontal examination records.

The diagnosis of periodontitis was based on radiographs taken within a 5-year period before and after delivery. Periodontitis is a chronic inflammatory disease that affects the tissues surrounding the teeth. Regardless of the disease stage, it is widely known that disease progression rates do not exceed 0.15 mm/year in terms of mean annual alveolar bone loss^43,44^. This indicates that the findings observed 5 years prior to or following delivery sufficiently reflect the patient’s periodontal status during pregnancy. The fact that no included subjects had ever been treated for periodontitis within the period between radiography and delivery makes our disease classification even more reliable.

A trained obstetrician and neonatologist collected clinical data from the mothers’ and neonates’ medical charts. Uterine leiomyoma was ultrasonographically diagnosed during the pre-pregnancy period or during pregnancy. SGA was defined as birth weight below the 10th percentile according to the Fenton growth chart^45^. The definitions of other variables are listed separately in the “Supplementary Material” (Supplementary Methods 1).

### Statistical analysis

SPSS version 20.0 statistical software package (SPSS, Inc., Chicago, IL, USA) was used for data analysis. After normality testing, the Mann–Whitney U test and Fisher’s exact test (two-sided) were used to compare continuous and categorical variables, respectively. Logistic regression analysis was performed after considering collinearity to analyse the predictors of SGA. SGA was the dependent variable, and the maternal variables with *P*-values lower than 0.1 in the Mann–Whitney U test or Fisher’s exact test entered in the regression model were the independent variables. Statistical significance was defined as a *P-*value < 0.05.

### Ethics statement

The study protocol was approved by the institutional review board (IRB) of the Korea University Anam Hospital (IRB No. 2020AN0128) with a waiver of informed consent. Patient records and information were anonymised and de-identified prior to data analysis. This study was carried out in accordance with the guidelines and regulations of the Declaration of Helsinki.

## Supplementary Information

Supplementary Information.

## Data Availability

The datasets generated and/or analysed during the current study are available from the corresponding authors upon reasonable request.
