# Supplementary material for: Radiological screening of maternal periodontitis for predicting adverse pregnancy and neonatal outcomes
Source: Sci Rep. 2020 Dec 4;10:21266. doi: 10.1038/s41598-020-78385-0 (PMC7718227; doi:10.1038/s41598-020-78385-0)
Supplement: Supplementary file 1 — Supplementary Information. [file 41598_2020_78385_MOESM1_ESM.docx]

**Title**

**Radiological screening of maternal periodontitis for predicting adverse pregnancy and neonatal outcomes**

**Authors:**

Ju Sun Heo,^1^ Ki Hoon Ahn,^2*^ and Jung Soo Park^3*^

^1^Department of Pediatrics, Anam Hospital, Korea University College of Medicine, Seoul, Republic of Korea

^2^Department of Obstetrics & Gynecology, Anam Hospital, Korea University College of Medicine, Seoul, Republic of Korea

^3^Department of Periodontology, Anam Hospital, Korea University College of Medicine, Seoul, Republic of Korea

* Ki Hoon Ahn and Jung Soo Park contributed equally as corresponding authors.

**Supplementary Fig. S1.** (a) Stage I or II periodontitis: Patients show no loss of any teeth due to periodontitis, and radiographic bone loss does not extend beyond coronal third of the root. (b) Stage III periodontitis: Alveolar bone loss extending to the middle third of the root, furcation involvement of class 2 or 3, and a vertical intrabony defect deeper than 3 mm, all which are evident on radiography and require an advanced form of periodontal treatment


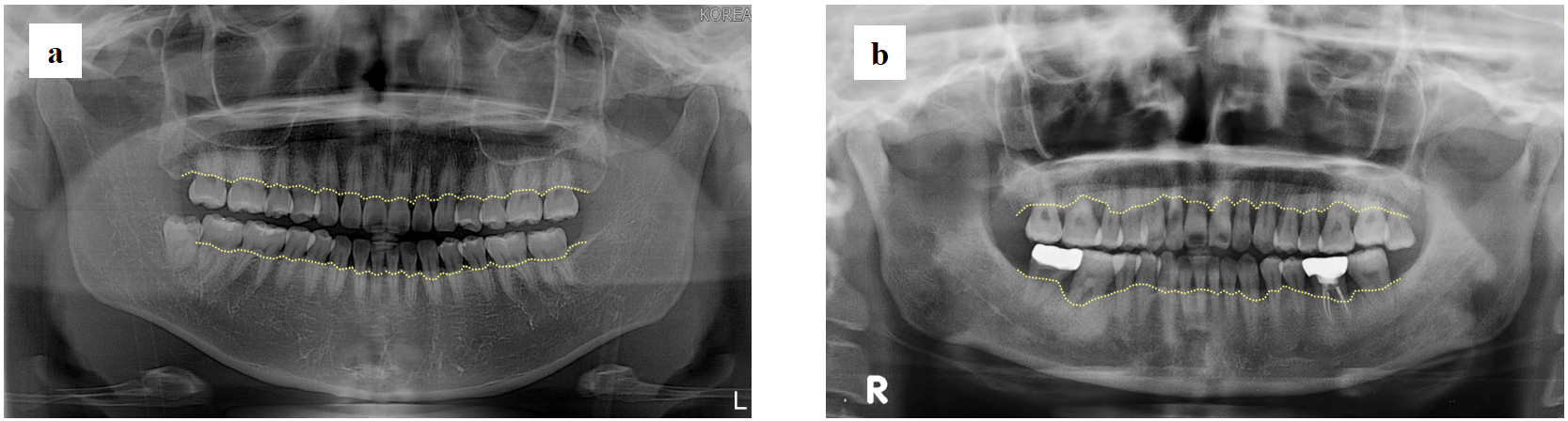


**Supplementary methods 1. The definitions of variables**

**Maternal factors**

Pre-pregnancy body mass index was calculated by dividing maternal pre-pregnancy weight (in kg) by height (in m) squared and is universally expressed in units of kg/m^2^. Data on maternal pre-pregnancy weight and height were collected via questionnaires during the first pre-natal check-up. Chorioamnionitis was histologically diagnosed. Placental histological examination findings for a variety of lesions (chorion, decidua, amnion and umbilical cord), including for histological inflammation, were reviewed by a specialised pathologist. Funisitis was defined as umbilical vasculitis with or without neutrophil infiltration into Wharton's jelly. Gestational diabetes mellitus was defined as glucose intolerance of variable degree with onset or first recognition during pregnancy, which was confirmed through a two-step strategy. The two-step strategy used 1-hour glucose tolerance test followed by a 3-hour oral glucose tolerance test (OGTT). An abnormal 3-hour glucose tolerance test result was defined by the presence of two or more of the following parameters: fasting glucose ≥ 95 mg/dL, 1-hour OGTT glucose level ≥ 180 mg/dL, 2-hour OGTT glucose level ≥ 155 mg/dL, and 3-hour OGTT glucose level ≥ 140 mg/dL. Chronic hypertension was defined as blood pressure exceeding 140/90 mmHg before pregnancy or before 20 weeks of gestation. Preeclampsia was defined as 1) hypertension in the latter part of pregnancy (> 20 weeks’ gestation) and proteinuria or 2) in absence of proteinuria, new-onset hypertension with the new onset of any of the following: thrombocytopenia, platelets <100,000/microliter; renal insufficiency, serum creatinine level >1.1 mg/dl or doubling of serum creatinine levels in the absence of other renal diseases; elevated liver transaminases (twice the normal concentration); pulmonary oedema; and neurologic abnormalities, unexplained new-onset headache unresponsive to medication, or visual symptoms. Chronic medical conditions included haemato-oncologic diseases (malignant lymphoma, aplastic anaemia, choriocarcinoma, prolactinoma), chronic kidney failure (renal transplantation), thyroid diseases (hyperthyroidism, hypothyroidism, thyroid cancer), autoimmune disease (systemic lupus erythematosus), liver diseases (liver cirrhosis, hepatitis), infectious diseases (tuberculosis, human immunodeficiency virus infection), and heart diseases (arrhythmia, congenital heart diseases).

**Neonatal factors**

Gestational age was determined according to the mother’s last menstrual period or using obstetric estimates. Z-scores of birth weight, height, and head circumference were calculated using the Fenton growth chart (1). Sepsis was defined as blood culture-positive sepsis accompanied by systemic antibiotic treatment for > 5 days. Respiratory distress syndrome was defined as a respiratory insufficiency that manifested at or shortly after birth, with radiologic findings compatible with respiratory distress syndrome. Moderate to severe bronchopulmonary dysplasia was defined as the need for supplemental oxygen or positive pressure ventilation at a postmenstrual age of 36 weeks, using the definition by the National Institute of Child Health and Human Development (2). Duration of respiratory support was defined as the total duration of invasive and non-invasive ventilation and supplemental oxygen therapy. Retinopathy of prematurity was defined using the criteria set forth by the International Committee for the Classification of Retinopathy of Prematurity (3). Necrotising enterocolitis was staged using modified Bell’s staging criteria (4). Intraventricular haemorrhage was classified as grade 1 or higher using the criteria by Papile et al (5).

**References**

1. Fenton, T. R. & Kim, J. H. A systematic review and meta-analysis to revise the Fenton growth chart for preterm infants. *BMC Pediatr.* **13**, 59 (2013).

2. Ehrenkranz, R.A., *et al.* Validation of the National Institutes of Health consensus definition of bronchopulmonary dysplasia. *Pediatrics*. **116**, 1353–1360 (2005).

3. Prematurity AICftCoRo. The International Classification of Retinopathy of Prematurity revisited. *Arch Ophthalmol*. **123**, 991–999 (2005).

4. Walsh, M. C., Kliegman, R. M. Necrotizing enterocolitis: treatment based on staging criteria. *Pediatr. Clin. North Am*. **33**, 179–201 (1986).

5. Papile, L. A., Burstein, J., Burstein, R., & Koffler, H. Incidence and evolution of subependymal and intraventricular hemorrhage: a study of infants with birth weights less than 1,500 gm. *J Pediatr*. **92**, 529–534 (1978).
